# Timeliness Vaccination of Measles Containing Vaccine and Barriers to Vaccination among Migrant Children in East China

**DOI:** 10.1371/journal.pone.0073264

**Published:** 2013-08-27

**Authors:** Yu Hu, Qian Li, Shuying Luo, Linqiao Lou, Xiaohua Qi, Shuyun Xie

**Affiliations:** 1 Institute of Immunization and Prevention, Zhejiang Center for Disease Control and Prevention, Hangzhou, P. R. China; 2 Institute of Immunization and Prevention, Yiwu Center for Disease Control and Prevention, Yiwu, P. R. China; Fudan University, China

## Abstract

**Background:**

The reported coverage rates of first and second doses of measles containing vaccine (MCV) are almost 95% in China, while measles cases are constantly being reported. This study evaluated the vaccine coverage, timeliness, and barriers to immunization of MCV_1_ and MCV_2_ in children aged from 8–48 months.

**Methods:**

We assessed 718 children aged 8–48 months, of which 499 children aged 18–48 months in September 2011. Face to face interviews were administered with children’s mothers to estimate MCV_1_ and MCV_2_ coverage rate, its timeliness and barriers to vaccine uptake.

**Results:**

The coverage rates were 76.9% for MCV_1_ and 44.7% for MCV_2_ in average. Only 47.5% of surveyed children received the MCV_1_ timely, which postpone vaccination by up to one month beyond the stipulated age of 8 months. Even if coverage thus improves with time, postponed vaccination adds to the pool of unprotected children in the population. Being unaware of the necessity for vaccination and its schedule, misunderstanding of side-effect of vaccine, and child being sick during the recommended vaccination period were significant preventive factors for both MCV_1_ and MCV_2_ vaccination. Having multiple children, mother’s education level, household income and children with working mothers were significantly associated with delayed or missing MCV_1_ immunization.

**Conclusions:**

To avoid future outbreaks, it is crucial to attain high coverage levels by timely vaccination, thus, accurate information should be delivered and a systematic approach should be targeted to high-risk groups.

## Introduction

Measles is a high contagious and potentially fatal viral infection which most commonly affects infants and young children. Although it is considered as one of the deadliest of all childhood fever/rash illness, it can be preventable by Measles Containing Vaccine (MCV), including measles vaccine, measles-rubella combined live attenuated vaccine and measles-mumps-rubella combined live attenuated vaccine. About 95% of single-dose recipients are considered to develop protective immunity against measles virus [1]. However, the remaining 5% of susceptible population is sufficient to sustain a measles outbreak, which is why many countries applied the two-dose MCV vaccination schedule in order to maintain a high level of vaccination coverage.

Recently, receiving the vaccination within a recommended age period has become an important issue in many countries. Numerous studies suggested that a certain proportion of the target population lacking timely vaccination could contribute a measles outbreak, even if the overall coverage of those people was high. Consequently, the importance of the recommended vaccination schedule has been reinforced in National Immunization Program (NIP) of many countries.

In 2006, the national committee for measles elimination of china executed the National Measles Elimination Plan (NMEP) [2], which implemented comprehensive strategies to satisfy the measles elimination criteria in 2012 issued by World Health Organization (WHO), including reinforcing the surveillance system of measles, conducting SIA, improving the coverage rate of MCV, and so on. Although China has made great progress in measles control in the framework of the NMEP, the average reported rate of measles was 28 cases per million in China in 2010, still far above the WHO’s recommended rate of one case per million. Similar to other countries [3], increasing population mobility coupled with low routine vaccination coverage of migrants has been identified as one of the key contributors of measles outbreaks in China [4]. The vaccination coverage of migrant children is much lower than that of local children [5], mainly as a result of migrants’ high mobility, low socioeconomic status, lower level of knowledge and awareness about vaccination, and insufficient access to vaccination services in receiving areas [6].

In the past decade, China had achieved remarkable economic growth through the development of market economy. Its GDP per capita increase almost 10 times from $721 in 1995 [7] to $6100 in 2011 [8]. However, regional income imbalance remained large. In 2011, annual incomes per capita for urban and rural households in eastern regions were 55% and 71% higher than those in western regions [8]. These regional disparities have become a key driving force of the country’s largest tide of internal migration in its history.

As a developed city in east areas in China, Yiwu city is located in east China and has a total area of 1105.5 Km^2^ with a population of 2.17 million residents. Yiwu’s is famous for small commodity trade and vibrant free markets and its rapid development has attracted 1 million migrants in 2011 [9], particularly from rural areas of China. According to official statistical data [9], 88% migrants came to Yiwu for work or business, 7% came just to be with their family members, and 5% came for other purposes. More than 90% migrants did not have their own houses in Yiwu: 70% lived in houses rented from the local people, and 20% lived in places provided by their employers. Due to high living cost in central of Yiwu, 90% of migrants lived in the suburban areas, especially in towns and townships with high density of population. Migrants lived in small single-storied dwellings with poor ventilation that accommodated one entire family and had no in house hygiene facilities. In this study, we aimed to discover the MCV coverage rate and its timeliness in children aged ≥8 months in Yiwu. We also aimed to identify the possible barriers to timely MCV vaccination.

## Methods

### Subjects

“Migrants” in our study refer to people living in an area without a temporary household registration card issued by Public Security Bureau of their current living areas [9]. Migrant children aged 8-48 months who had lived in the surveyed areas continuously for 1 month or more at the time they were interviewed were selected as study subjects. The main reason for this choice was migrant children who had lived in their catchment areas continuously for 1 month or more should to be identified and immunized by health facilities in Yiwu city according to the Ministry of Health’s(MoH) immunization guideline [10]. We documented the date of the last immigration to the surveyed areas of the migrant children in our interview, so we can identify the length of time that the migrant children continuously lived in the surveyed areas.

### MCV immunization schedule and measurements of timely vaccination

Health facilities in Yiwu adopt the primary vaccination schedule recommended by the MoH [10], which stipulates all children should get one dose measles-rubella combined live attenuated vaccine as the first dose of MCV(MCV_1_) at 8 month of age, and get one dose of measles-mumps-rubella combined live attenuated vaccine as the second dose of MCV(MCV_2_) during 18~24months. We evaluated the timeliness of MCV_1_ for which the recommended vaccination age period is from the first day to the final day of the 8th month of life and MCV_2_ for which the recommended vaccination age period is from the first day of 18th to the final day of the 24th month of life. In addition, Since parental recall of their child’s immunization history was unreliable, only written vaccination history(immunization cards) was accepted as the proof of timely vaccination or evaluating possible factors related to delayed or missed vaccinations in our study.

### Sampling and survey

The demographic data of each 13 town of Yiwu city were collected and collated by the EPI staff of Yiwu Center for Disease Control and Prevention(CDC). Those villages where migrant people accounted for more than 50% of the general population were selected as our investigation sites. Finally, a total of 66 villages were selected. The sampling method was based on the WHO’s Advocated Cluster Sampling Technique Manual [11]. Coverage rates for MCV_1_ and MCV_2_ of children aged 8 months-4 years were assumed to be around 70% according to the pilot survey done before and the desired precision was 5%. We assumed a design effect of 2 and obtained required number of surveyed children per cluster for variable numbers of clusters through the table recommended by WHO manual. Ten children per cluster for 66 clusters were finally determined as our sample size.

We got household list of each selected resettlement colony from local administrative office and randomly selected one household(using random numbers) as the first family of an eligible child to be interviewed, and continued by choosing each subsequent household located to the right of the previous one until ten eligible children were interviewed. Only one child per household was selected to avoid clustering. When two or more eligible children were in the same household, the youngest child was selected based on the WHO manual.

Twenty-six EPI staff of Yiwu CDC were selected and trained as interviewers. We hold a training meeting for all the field interviewers. We introduced the background of this investigation, sampling method, the contents of the questionnaire(See Appendix A) and exact meaning of each choice, quality control, investigation skills for sensitive question etc. All of the interviewers practice the interviews in the training meeting to be familiar with the questionnaire and improve the interview skills. Local guides were selected from public health liaisons(some private doctors who are recruited by village level administration and help the health center in town level to deliver the basic public health service package) of surveyed villages to familiarize interviewers with local circumstances and introduce interviewers to the surveyed household. Both household and clinic surveys within each town should be completed within one day. During each visit of the household with eligible children, standard face-to-face interviews were conducted with mothers to evaluate the vaccination coverage status and factors that may have been significantly associated with a delayed or missed vaccination dose.

Vaccination status and age at vaccination were confirmed by checking the immunization card kept by mothers or the local health facilities. When the immunization card was not available, vaccination status was determined by information from the mothers, according to recall. The information on whether the child had been vaccinated, the date of vaccination, and the health facility at which the child was vaccinated were collected. We also adopted the measurements mentioned previously to evaluate the timeliness of vaccination.

The Protection Motivation Theory Model(PMT) [12] was applied to evaluate possible factors related to delayed or missed vaccinations. In the PMT, whether a person practices a particular health practice could be attributed to two major factors: perception of susceptibility to an illness, and the perception of benefit resulting from a health practice. In addition to susceptibility and benefit, we evaluated barriers to immunization, e.g. cost, lack of time, and accessibility. Mothers were also tested on their knowledge of measles by ten questions. Demographic and socioeconomic factors were also surveyed. PMT scores were calculated in five categories: susceptibility to measles(caregivers’ perception on the most susceptible sub population for measles infection), severity of measles(caregivers’ perception that measles is a severe disease or not), benefit of vaccination(caregivers’ perception on the MCV vaccination as an effective prevention for measles), barriers to vaccination(caregivers’ perception on cost of vaccination, adequate vaccination service nearby, and convenience of opening time of health facility), and knowledge about MCV vaccination(caregivers’ perception on EPI policy, schedule of MCV, effect of MCV, precaution of MCV vaccination, and so on). Susceptibility, severity, benefit, and barriers to vaccination were presented as an average of scores that range from 0 (not at all) to 5 (very much). Knowledge of measles vaccination was worth 1 point for each correct answer, the total knowledge score ranged from 0 to 10.

### Statistical analysis

Description analysis and χ^2^ test were used to present the vaccination status among different age groups. Survival analysis by the Kaplan-Meier method was performed to present the timeliness vaccination and cumulative coverage of MCV, and log-rank test was used to test for differences among cohorts. It seemed that survival analysis using the Kaplan-Meier method would give more exact coverage figures than standard calculations. It should be the method used when following vaccination coverage over time and we have later discovered that this method was also recently suggested by Laubereau [13]. Kruskal-Wallis test was used to compare the PMT scores in timely, delayed, and unvaccinated groups. Multivariate logistic regression was used to evaluate the association between various social factors and vaccination status. All the analyses above applied Statistics Package for Socio Science (SPSS) software, version 13.0.Statistical significance was defined by a *P* value of <0.05.

### Ethnical considerations

This study was approved by the Ethical Review Board of Zhejiang Provincial Center for Disease Control and Prevention. In each household surveyed the informed consent form was discussed with the parents or legal representatives of the child, and signed by one of them once there was a decision to participate.

## Results

### Coverage and timeliness

Vaccination status for MCV_1_ and MCV_2_ were collected from a total of 718 children aged from 8 months to 48 months old, of whom 52 (7.2%) had no written vaccination history(immunization cards) and date of vaccination. The numbers of children surveyed were 108 aged 8-12 months, 213 aged 13-24 months, 209 aged 25-36 months, 188 aged 37-48 months, and 102 aged 18-24 months, respectively for each cohort. The coverage rates were 76.9% for MCV_1_ and 44.7% for MCV_2_ in average. When vaccination according to mothers’ recall was taken into account, the coverage rate was increased by 2.6% and 6.6%, respectively. Some 20% and 48% of the children will never be vaccinated for MCV_1_ and MCV_2_, respectively (Table 1).

**Table 1:** Vaccination coverage rate in surveyed children, Sep, 2011.

Age	Total	Immunization cards			By recall
		Vaccinated	Unvaccinated		
MCV_1_^*^	718	552 (76.9%)	147 (20.5%)		19 (2.6%)
8-12 months	108	96 (88.9%)	12 (11.100%)		0 (0.0%)
13-24 months	213	154 (72.3%)	54 (25.4%)		5 (2.3%)
25-36 months	209	157 (75.1%)	44 (21.1%)		8 (3.8%)
37-48 months	188	145 (77.2%)	37 (19.7%)		6 (3.2%)
MCV_2_^#^	499	223 (44.7%)	243 (48.7%)		33 (6.6%)
18-24 months	102	58 (56.9%)	39 (38.2%)		5 (4.9%)
25-36 months	209	92 (44.0%)	106 (50.7%)		11 (5.3%)
37-48 months	188	73 (38.8%)	98 (52.2%)		17 (9.0%)

*:χ^2^=14.1, *P*=0.028; #:χ^2^=10.5, *P*=0.033.

**Figure 1:**
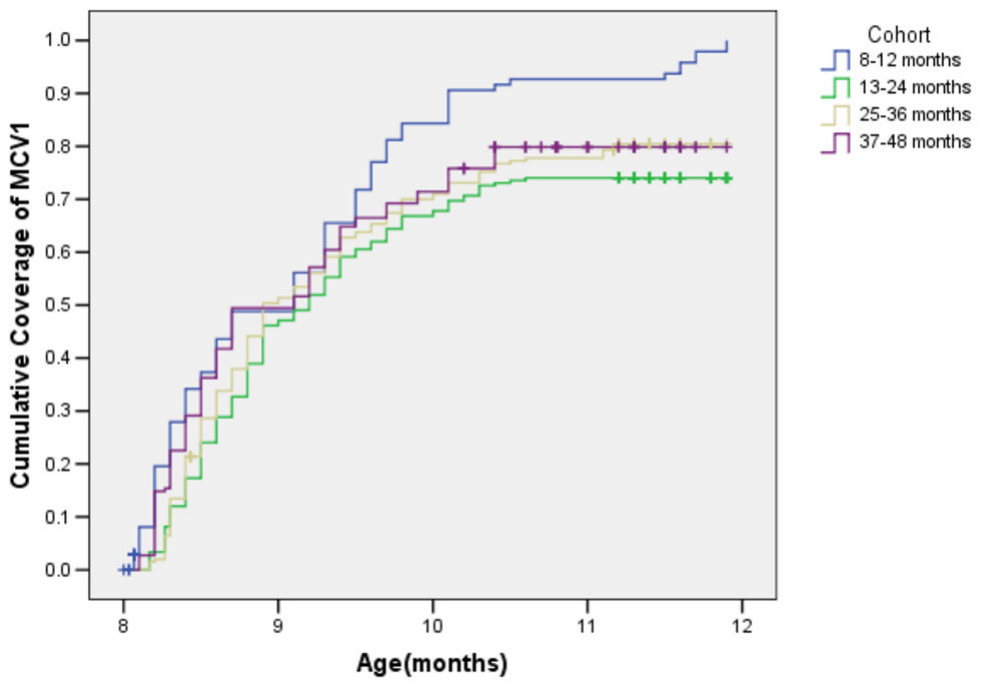
Cumulative coverage of MCV_1_(%) for different cohorts. According to immunization cards by month of age in the four cohorts of children aged 8-12 months(blue line), 13-24 months(green line), 25-36 months(yellowe line) and 37-48 months(purple line). Number of children in each cohort is 108, 208, 201 and 182, respectively.

Figure 1 showed the cumulative coverage of MCV_1_ in each cohort as a function of age(by months). Timely vaccination rates for the MCV_1_ whom immunization cards were available were lower than the overall coverage. Of the 699 children aged 8-48 months, 332 (47.5%) received the MCV_1_ at 8 month of age. The median age of MCV_1_ vaccination was at 9.1 months of age(95% of Confidence Interval, 95%CI: 8.9-9.2). Compared with the elder age group, the MCV_1_ coverage rates of lower children were significantly higher(*P*=0.000 by log-rank test).

### Barriers to timely vaccination

**Figure 2:**
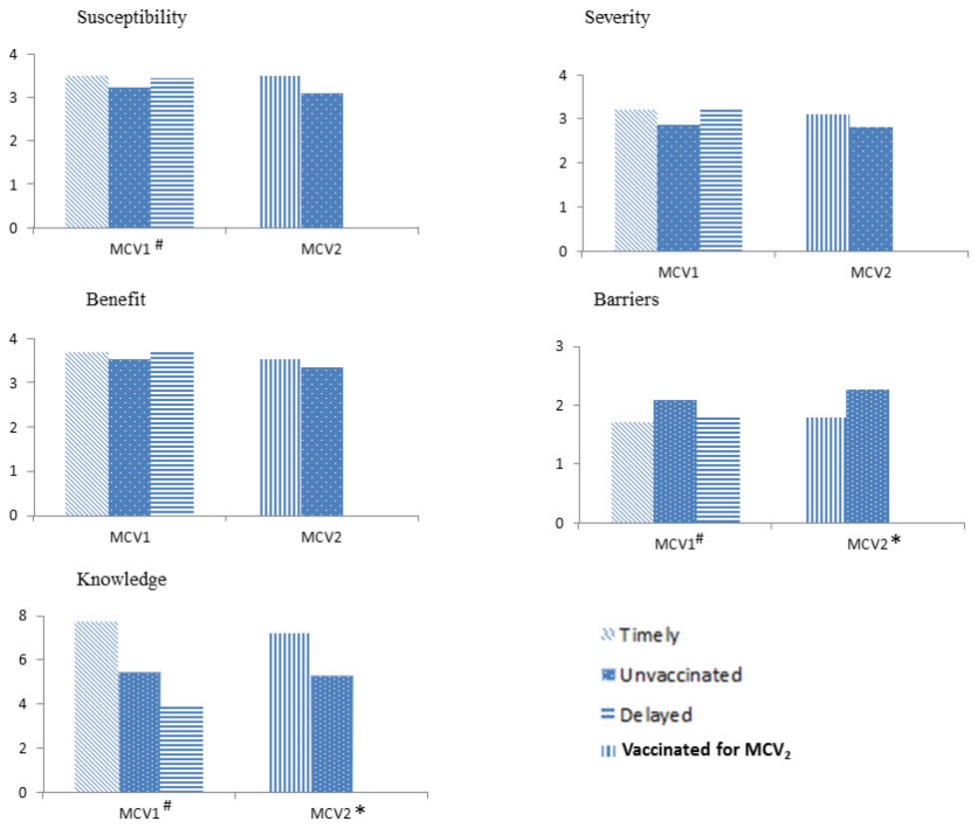
Scores of PMT model for MCV_1_ and MCV_2_. ^#^Significant difference among three groups(*P*<0.05). *Significant difference between two groups(*P*<0.05).

Analysis of the PMT model revealed that for MCV_1_, the average score of barriers of vaccination was significantly higher in the unvaccinated group than that in the delayed and timely groups, while the average scores for perception of susceptibility and knowledge were significantly lower in the unvaccinated group than those in the delayed and timely groups. For MCV_2_, the average score of barriers was significantly higher in unvaccinated group than that in vaccinated group, while the average score of knowledge was significantly lower in unvaccinated group than that in vaccinated group. Perception of severity and benefit were not significantly associated with missed or delayed MCV doses(Figure 2).

Be aware of necessity for vaccination and its schedule were significantly associated with both MCV_1_ and MCV_2_ vaccination. Mothers in the unvaccinated group for both MCV_1_ and MCV_2_ group were more likely to have an erroneous impression that measles immunity could be achieved naturally. Of MCV_2_ vaccination group, 17.3% of the mothers who had not had their child vaccinated stated that the reason was concern about the side-effect of vaccination. Child being sick during the recommended vaccination period was also a significant factor for both MCV_1_ and MCV_2_ vaccination(Table 2).

**Table 2:** Preventive vaccination factors of the PMT model and MCV vaccination.

Factors	MCV_1_(%)			MCV_2_(%)	
	Timely (n=332)	Delayed (n=220)	Unvaccinated (n=147)	Vaccinated (n=223)	Unvaccinated (n=243)
I did not know the vaccination was necessary^*^	1.2	4.5	32.7	3.6	23.0
I did not know the immunization schedule^*^	5.1	10.9	41.5	6.7	29.6
I thought immunity could be achieved without vaccination^$^	1.2	3.2	12.2	2.2	6.9
I thought vaccine was not effective	2.4	2.3	3.4	2.2	2.5
I concerned about the side-effect of vaccination^#^	9.6	9.5	18.4	8.2	17.3
I thought measles was not serious	3.3	3.6	6.1	3.6	4.5
Adequate vaccination service was not accessible	2.7	3.2	4.8	2.7	3.3
Vaccination was costly	0.6	1.6	2.7	1.3	3.3
I was not able to visit the health facility during its opening time	6.9	9.1	12.2	7.2	10.7
Child was sick during the recommended period^*^	5.4	36.8	11.6	4.0	9.8

*: *P* <0.05 for both two groups. $: *P* <0.05 for MCV_1_, Fisher’s Exact Test *P*=0.012 for MCV_2_. #: Fisher’s Exact Test *P*=0.012 for MCV_2_ only

In the multivariate logistic regression analysis, we found those factors including having multiple children, mother’s education level, household income were significantly associated with delayed or missing MCV_1_ immunization. Children whose mother have a job were almost 3 times higher to be unvaccinated than children with a non-working mother(Table 3).

**Table 3:** Multivariate analysis for MCV_1_ timeliness vaccination by various variables.

	Delayed						Unvaccinated					
	COR	95%CI	*P*	AOR	95%CI	*P*	COR	95%CI	*P*	AOR	95%CI	*P*
Multiple children			<0.05			<0.05			<0.01			<0.01
One^#^	-	-		-	-		-	-		-	-	
Two	0.8	0.6-1.7		1.3	0.7-2.9		4.8	2.6-8.3		2.2	1.2-5.1	
Three or more	1.8	1.1-2.6		1.6	1.2-3.3		6.1	3.7-12.9		2.7	1.8-6.3	
Mother’s age			<0.05			>0.05			<0.05			>0.05
<30^#^	-	-		-	-		-	-		-	-	
≥30	1.4	1.2-2.6		1.1	0.4-1.7		2.6	1.6-7.3		1.3	0.6-3.2	
Mother’s education level			<0.01			<0.01			<0.01			<0.01
Under high middle school education^#^	-	-		-	-		-	-		-	-	
high middle school and above	2.6	1.5-3.7		3.1	2.2-5.8		5.9	3.5-10.7		3.5	2.5-6.7	
Mother’s job^#^			<0.05			>0.05			<0.01			<0.01
Yes	-	-		-	-		-	-		-	-	
No	0.5	0.2-0.8		1.1	0.4-3.3		0.5	0.3-0.8		0.3	0.1-0.9	
Household income(monthly)			<0.01			<0.05			<0.01			<0.01
High(>4000 CNY)^#^	-	-		-	-		-	-		-	-	
Average(2000-4000 CNY)	1.7	1.3-2.2		0.9	0.2-2.4		4.6	3.1-8.5		1.1	0.4-3.1	
Low(<2000 CNY)	5.3	3.3-8.6		3.5	1.7-6.9		7.3	3.3-18.6		5.1	2.1-9.3	

^#^ Reference group.

COR: Crude Odds Ratio; AOR: Adjusted Odds Ratio; *CI*: Confidence Interval

## Discussion

Many of previous studies on measles vaccination have focused on the accumulative uptake rate for a certain age. However, relying on the overall vaccination coverage may be crude, which may mask the substantial delays in vaccination and subsequent lack of immunity. Hence, there could be a measles outbreak in the highly vaccinated population which was attributed to vaccine failure resulted from individuals being vaccinated outside the recommended period [14]. In our study, we analyzed the timeliness of MCV_1_ which revealed that only 47.5% (332/699) of surveyed children with immunization cards were immunized during the recommended age period which left about more than half of children either delayed or missing immunization.

Still assuming that maternal antibodies disappear at 6 months on average, this means that each child is now susceptible to measles for close to 2 months. This period is also probably lengthening, since the children who were first started in the NIP in 1978 in China, are now themselves becoming parents, and since several studies [15–17] indicate that children to vaccinated mothers lose their maternal antibodies earlier than children whose mothers had the natural infection. Thus, several studies point to the timeliness of MCV_1_ vaccination being of great importance, and render that lacking of timely vaccination is likely to be one of many causes of measles epidemics. The main reason for this is that postponed vaccination adds to the proportion of children unprotected in population. For example, the investigation of a measles epidemic in the USA during 1989-1991 concluded that “only a sustained effort to provide age-appropriate vaccination will prevent another resurgence of measles” [18], since the primary cause for the epidemic was failure to provide vaccines on schedule [19]. Also, in Germany, Siedler [20] came to the conclusion that a considerable reduction in measles incidence would be achieved if MCV was delivered according to the recommended schedule.

Another implication is that children who were delayed or unvaccinated in MCV probably had difficulties with medical access and did not catch up their vaccination schedule even at a later age. These children also had insufficient utilization of other preventive and primary care such as screening, acute illness visits and well-child care visits. Hence, increasing healthcare access for vaccination is likely to increase use of other primary health service.

Our study revealed that caregivers who did not have their child vaccinated were the least aware of the necessity for vaccination and its schedule for both MCV_1_ and MCV_2_. Another reason for delayed or missed vaccinations was that the child was sick during the recommended immunization period. However, because postponing the vaccination was a voluntary decision by the caregiver, not made by a doctor, we can infer that accurate information about vaccination is not adequately delivered to the general public and that insufficient knowledge is related to inadequate vaccination [21]. Thus, interventions should be implemented to improve the knowledge level and avoid misunderstandings. Other interventions on addressing the needs of these mothers should also be included, such as extending office hours of health facilities, and attitude changes allowing fathers to share the responsibility as well. What is more, education and information delivery should be coupled with changes in social environment and structure in order to promote vaccination coverage and timeliness.

A second dose of MCV is administered in order to induce immunity in those who failed to attain immunity through MCV_1_. After the new school entry vaccination requirement was implemented in China in 2005, the overall coverage rate of MCV_2_ 6-year-old children, which is the age of primary school entry, was reported to be almost 95% [22]. Our study revealed that the rate of MCV_2_ of children aged 8-48 months with immunization cards was only 47.8%. It can be speculated that although it appears almost all children of school age have been vaccinated, many of these children are not vaccinated on time. Several reasons could be responsible for this untimely vaccination of MCV_2_. First, the relatively long range of the recommended period gives the sense that there is still plenty of time left for vaccination. This may apply not only mothers but also to doctors, subsequently resulting in delay of vaccination for relatively minor health problems of the child [23]. Second, as most of the childhood vaccination schedule is completed around the first year of life, mothers are less likely to pay attention to MCV_2_ which is scheduled at18-24 months of age. Modifying the current school-age vaccination requirement to an earlier age and developing and performing reminder(due soon) and recall(past due) systems to improve the coverage rate and timeliness of MCV which has been found to be effective in increasing attendance at health facilities and improving vaccination rates in various settings [24].

Our study suggests that having more than one children is a significant demographic factor for delayed or missed MCV_1_. Several studies have suggested that parental attention can be diverted by the presence of multiple children [25,26].

Having a mother with a higher level of education and a job were significant demographic factors for delayed or missed vaccination for MCV_1_. As mothers with higher education are more likely to have a career, we assume that they have less time to spare for their child’s primary healthcare and at the same time, are less aware of the necessary information concerning vaccination. It may suggest that higher education of the mother does not necessarily correlate with positive health behaviour related to vaccination.

Not many studies have evaluated the association of economic status with vaccination timeliness. Mothers in our study who had lower economic status were more likely to delay or miss vaccination to their child than those who had a middle or higher economic status. This result is in similar to studies [26,27] performed in other social circumstances where using a public health vaccination provider was associated with delay in vaccination. We assumed that it may be associated with mothers’ misunderstandings, such as “vaccination was costly” coupled with “vaccination was unnecessary”(spearman correlation coefficient was 0.89, *P*<0.001).

This study is subjected to several limitations. First, recall bias may occurred due to partly vaccination coverage was provided from mothers who did not have immunization cards by their memory. In order to evaluate the magnitude of possible recall bias, we presented the proportion of coverage by recall. It was revealed that only 7% had no written history of immunization, suggesting that the extent of bias is not significant. Second, we did not collect data on health facilities’ supply and other manpower, infrastructure-related issues and thus cannot draw any conclusions on health care providing system. The strength of our study is that it is a community-based study. Moreover, it is one of few studies performed in the dense population coupled with concentrated migrant area that evaluates the timeliness of, and barriers to, MCV vaccination.

## Conclusion

The timely vaccination coverage rate of MCV in our study was suboptimal and promotion of timely vaccination needs to be encouraged. Accurate information should be delivered especially migrant children’s caregivers. Children who have siblings should be targeted as high-risk subgroups. Health education and information delivery should be coupled with changes in social environment and structure in order to promote vaccination coverage and timeliness. A systematic approach, such as lowering the vaccination requirement to a younger age might also be encouraged.

## Supporting Information

Table S1 **EPI questionnaire for migrant children and their mother is shown.**
(DOCX)
