# Supplementary material for: Timeliness Vaccination of Measles Containing Vaccine and Barriers to Vaccination among Migrant Children in East China
Source: PLoS One. 2013 Aug 27;8(8):e73264. doi: 10.1371/journal.pone.0073264 (PMC3755000; doi:10.1371/journal.pone.0073264)
Supplement: Table S1 — EPI questionnaire for migrant children and their mother is shown. (DOCX) [file pone.0073264.s001.docx]

Supporting information file:

Table S1 EPI Questionnaire for Migrant Children and Their Mother

**Introductory note:**

For dear migrant child as well as mother: This following questionnaire is about migrant child’s immunization situation and its potential determinants. It will help us to improve the immunization service. Thank you for your participation.

**Part 1 Code for questionnaire**

| 1. Standard county No.: | □□□□□□ |
| --- | --- |
| 2. Resettlement colony No.： | □□ |
| 3. Target child No.： | □□ |

**Part 2 Family and child’s information**

| 4. Name of surveyed child: _____________ | | |
| --- | --- | --- |
| 5.Gender of the child: (1) Male (2) Female | | |
| 6.Child’s birthday(yyyy/mm/dd): | | □□□□□□□□ |
| 7. Ethnic of child: (1) Han (2) Minority ethnic | | □ |
| 8.Place of delivery(where did the child born?) : (1) Hospital (2) Home | | □ |
| 9.When was the last immigration to the surveyed areas of child?  (yyyy/mm//dd): | | □□□□□□□□ |
| 10. Mother’s name: _____________Cell phone：_____________ | | |
| 11. Mother’s age: (1) <30 (2)≥30 | | □ |
| 12.Mother’s education level: (1) Under high middle school education  (2) High middle school and above | □ | |
| 13.Do mother have a job? (1) Yes (2) No | □ | |
| 14.When was the last immigration to the surveyed areas of child?  (yyyy/mm//dd): | | □□□□□□□□ |
| 15.How many children do the family have? | | □ |
| 16.Place of delivery(where did the child born?) (1) Hospital (2) Home | | □ |
| 17. Household monthly income(CNY)  (1) High: >4000 (2) Average: 2000-4000 (3) Low: <2000 CNY | | □ |

**Part 3 MCV vaccination information**

| MCV | Dose | Vaccinated date  (yyyy/mm/dd) | Child was sick during the recommended  period: (1) yes (2)no |
| --- | --- | --- | --- |
|  | 1 | □□□□□□□□ | □ |
|  | 2 | □□□□□□□□ | □ |

**Part 4 Mother’s attitude and awareness towards MCV immunization**

| 18. Which group is the most susceptible for measles infection?  (1): Newborns and infants  (2): Pre-school children  (3): School-age children and adolescent  (4): Adult  (5): Elderly people | □ | |
| --- | --- | --- |
| 19. Is measles a severe disease?  (1): It is very severe and it can cause death if we do not treat timely.  (2): It is a severe disease but it need hospitalization.  (3): It is a mild disease it can recover well under medical treatment .  (4): It is not serious and it can recover well even without any medical treatment.  (5): I do not know. | □ | |
| 20. Do you consider that vaccination was costly?  (1): It is cost a lot money to get all vaccine which was introduced by immunization physicians  (2): It cost a lot of money but I can afford  (3): It cost money but not lot  (4): Some vaccines are free of charge, but I could tell the details  (5): All the vaccine are free of charge | | □ |
| 21. Could you get adequate vaccination service near your home?  (1): No, I do not know where I can get vaccination service  (2): Most of time I can get adequate vaccination service, but I have met the situation that should waiting for more than one hour  (3): I can get adequate vaccination service, but I have met the situation that should waiting for more than half an hour  (4): I can get adequate vaccination service and I just wait less than 15 minutes | | □ |
| 22. Is the opening time of health facility fit for you and your children?  (1): No, I was not able to visit the health facility during its opening time  (2): Sometimes, the health facility was closed when I visited  (3): There is no conflict between health facility opening time and my spare time | | □ |
| 24. Do you know the free of charge policy for MCV vaccination?  (1): Yes  (2): No | | □ |
| 25. Do you know that every child should get two dose of MCV according to the schedule of national immunization program?  (1): Yes  (2): No | | □ |
| 26. Do you know the antibody after MCV vaccination can be effective for a life-long time?  (1): Yes  (2): No | | □ |
| 27. Do you know the 1st dose of MCV should get at the 8th month of age?  (1): Yes  (2): No | | □ |
| 28. Do you know the 2ed dose of MCV should get at the 18th month of age?  (1): Yes  (2): No | | □ |
| 29. Do you know each child should keep the immunization card carefully as the certificate for vaccination?  (1): Yes  (2): No | | □ |
| 30. Can you tell us a specific kind of MCV?  (1): Yes  (2): No | | □ |
| 31. If your child gets a fever on the scheduled day for MCV, Is it proper for MCV vaccination?  (1): Yes  (2): No | | □ |
| 32. Do you know the opening time of the health facility near home?  (1): Yes  (2): No | | □ |
| 33. Do you know the due date for the next vaccination?  (1): Yes  (2): No | | □ |

Interviewer Signature: ___________ Qulity control Signature： ___________

Signature Date: ___________ Signature Date: _____ ______
